# Engagement in HIV Medical Care and Technology Use among Stimulant-Using and Nonstimulant-Using Men who have Sex with Men

**DOI:** 10.1155/2013/121352

**Published:** 2013-06-23

**Authors:** Keith J. Horvath, Adam W. Carrico, Jane Simoni, Edward W. Boyer, K. Rivet Amico, Andy E. Petroll

**Affiliations:** ^1^Center for AIDS Intervention Research, Medical College of Wisconsin, 2071 N. Summit Avenue, Milwaukee, WI 53202, USA; ^2^School of Nursing, University of California, San Francisco 2 Koret Way, San Francisco, CA 94143, USA; ^3^Department of Psychology, University of Washington, Box 351525, Seattle, WA 98195-1525, USA; ^4^Department of Emergency Medicine, University of Massachusetts Medical School, 55 Lake Avenue North, Worcester, MA 01655, USA; ^5^Center for Health, Intervention and Prevention, University of Connecticut, 2006 Hillside Road, Unit 1248, Storrs, CT 06269-1248, USA

## Abstract

Aims of this study were to assess the associations between stimulant use and attitudes toward and engagement in HIV medical care and to examine technology use among stimulant-using and nonstimulant-using men who have sex with men (MSM). HIV-positive MSM (*n* = 276; mean age = 42 years; 71% white, non-Hispanic; 43% with college degree) completed an online survey in 2009. Most men (69%) had not missed any scheduled HIV medical appointments in the past year, while 23% had missed at least one, and 9% had not attended any appointments. Stimulant use was significantly associated with not attending any HIV medical appointments in the unadjusted model (relative risk ratio (RRR) = 2.84, 95% CI [1.07, 7.58]), as well as in models adjusted for demographic (RRR = 3.16, 95% CI [1.13, 8.84]) and psychosocial (RRR = 3.44, 95% CI [1.17, 10.15]) factors (*Ps* < 0.05). Fewer stimulant-using than non-stimulant-using men rated HIV medical care a high priority (57% versus 85%; *P* < 0.01). Few significant differences were found in online social networking or mobile phone use between stimulant-using and non-stimulant-using MSM, even when stratified by engagement in HIV care. Findings indicate that stimulant use is uniquely associated with nonengagement in HIV medical care in this sample, and that it may be possible to reach stimulant-using MSM using online social networking and mobile technologies.

## 1. Introduction

Studies show that engagement in HIV medical care is challenging for many persons with HIV infection [1, 2]. Just over three quarters (77%) of persons who are aware that they have HIV are estimated to be linked to HIV care in the USA; even fewer (51%) who are aware that they have HIV are estimated to be retained in HIV care [3]. Illicit drug use is a significant risk factor for poor engagement in HIV medical care [4]; however, the specific influence of stimulant use on retention in HIV care is not presently well understood, despite its pervasive use in one of the populations most heavily burdened by HIV infection—men who have sex with men (MSM). Stimulants are a class of drugs known to produce a sense of euphoria and increase sexual arousal [5] and include methamphetamine, amphetamine, cocaine, and MDMA (“ecstasy”) [6]. In a study of demographic and psychosocial factors associated with stimulant use among 711 MSM living in San Francisco in 2002-2003, the prevalence of any stimulant use in the past 6 months was 23% [7]. In that study, the most commonly reported stimulant was methamphetamine (17% of the sample), followed by powder cocaine (10%), crack cocaine (2%), and amphetamine (1%). Younger age, HIV-positive status, depressed mood, and sexual compulsivity were associated with any stimulant use in the past 6 months [7]. Studies of HIV-positive MSM in USA show that methamphetamine use ranges from approximately 10% to 32% [8–12]. Taken together, current evidence suggests that stimulant use is prevalent among HIV-infected MSM.

Stimulant use among people with HIV has been associated with more rapid disease progression, including faster progression to AIDS, development of AIDS defining illnesses, and hastened AIDS-related mortality [13]. Studies consistently show that stimulant use is associated with decreased odds of antiretroviral therapy (ART) utilization, poorer ART adherence, difficulties with ART persistence (i.e., duration of time continuously on ART), and elevated HIV viral load [13–20]. In the era of HIV treatment as prevention (TasP), innovative approaches are needed to promote engagement in HIV care among stimulant users in order to achieve sustained viral suppression and decrease the likelihood of onward HIV transmission to their uninfected sexual partners [21].

Few, if any, studies have examined the association between stimulant use and poor engagement in HIV medical care. In interviews with 20 HIV-infected MSM who reported that they seroconverted in the context of methamphetamine use, 60% of men reported that methamphetamine use compromised their self-care behaviors, including attending medical appointments [22]. The most common reasons reported by men were that methamphetamine use caused them to forget to engage in self-care behaviors and that it reduced their motivation to engage in these activities. Although more research needs to be conducted to assess attitudinal barriers to engagement in HIV care among stimulant users, at least one study of barriers to attending drug treatment among stimulant users living in the Southern USA showed that a relatively low proportion of participants perceived the need for treatment (19%). Engagement in substance abuse treatment has a number of similarities with engagement in HIV medical care, including the prioritization of health behaviors and the potential for disruption of one's regular routine.

Stimulant-using persons may be less likely to prioritize their HIV care because of the acute effects of substance use or withdrawal, mental health comorbidities, or chaotic life circumstances [23, 24]. An emerging innovation in providing critical behavioral intervention messages or components in natural environments to hard-to-reach patient populations is to use available mobile and social networking technologies. It is estimated that 67% of USA Internet-using adults use social networking sites, and 46% of USA adults own a smart phone [25, 26]. Technology-based interventions for people with HIV have proliferated in recent years, including the use of mobile phone counseling, text messaging and interactive computer-based programs [27]. A recent study showed beneficial impacts of text messages to reduce methamphetamine use and sexual risk behavior among methamphetamine-using MSM [28]. 

Despite the potential reach and impact of technology to promote retention in HIV care among persons where disorganization and challenges to prioritization of self-care may be particularly acute, little (if any) information is available about social networking site and mobile phone use among stimulant-using persons with HIV. Technology-based intervention approaches to promote engagement in HIV care, and other aspects of self-care would offer unique efficiencies in reach and coverage to the extent that the targeted population actually use these technologies. The purpose of this study with HIV-positive MSM was to (1) assess the associations of stimulant use with attitudes toward HIV medical care; (2) examine whether recent stimulant use is associated with poorer HIV medical care engagement, and (3) compare social networking site and mobile phone use between stimulant-using and nonstimulant-using MSM. 

## 2. Methods

### 2.1. Participant Recruitment

Participants responded to an online survey between July and November, 2009. Inclusion criteria to participate in the online survey were 18 years of age or older, English speaking, self-reported HIV-positive status, and United States residence. A total of 387 participants completed the online survey. For the purpose of this study, the following exclusion criteria were established: 58 were excluded for not being born biologically male, 16 were excluded for not self-identifying as gay or bisexual, 36 were excluded for being diagnosed with HIV within 1 year of answering the survey (as HIV medical appointment engagement was assessed for the past 12 months), and 1 was excluded for not providing sufficient data. Thus, the final dataset used for the purpose of this study included 276 HIV-positive MSM.

### 2.2. Study Procedures

Study procedures are described in greater detail elsewhere [29]. Briefly, participants were recruited in several ways: (1) 53.5% were recruited from banner advertisements on, or e-mail newsletters from, HIV-related websites; (2) 12.8% were recruited from targeted ads on *Facebook*; (3) 4.5% were recruited from an e-mail sent to men who had participated in a prior study by the research team and requested to be notified of future research opportunities; (4) 1.6% were recruited from an online search; 27.6% were recruited from fliers and postcards at AIDS Service Organizations (ASOs) with information that directed participants to the study website. Regardless of how participants were recruited, they must have had a valid e-mail address to access the screening questionnaire, and they must have completed the survey online. Multiple security measures were used to block repeated attempts to screen for eligibility, including cookies placed in browsers, e-mail address screening, and IP address screening. Participants who were deemed valid and completed the survey were reimbursed $25 for their time.

### 2.3. Measures

#### 2.3.1. Demographic Variables

Demographic characteristics examined for the purpose of this study included age (in years); education (less than 8th grade; 8th to 11th grade; high school graduate/GED; technical school; some college or associates degree; college bachelor degree; graduate or professional degree); race (African American, white, Asian or Asian American; American Indian or Alaskan Native; or Native Hawaiian or other Pacific Islander); ethnicity (Hispanic/Latino); and years living with HIV. Participants were asked to self-report the result of their most recent CD4+ count with the question: “T-cell (also called CD4+) count usually ranges from 0 to 1600 cells/mm^3^. What was your most recent T-cell (CD4+) count?” 

#### 2.3.2. Psychosocial Variables

Psychosocial variables used in this study were depression, life chaos, and alcohol use. Depression was measured with the 10-item *Center for Epidemiologic Studies*-*Depression* Scale (CES-D) [30], a widely used measure of depression in research studies (*α* = 0.88 for this sample of MSM). A cut-off score of 10 or higher was used to indicate significant depressive symptoms [30, 31]. 

The *Life Chaos* Scale is a 6-item measure of whether someone has a stable and predictable lifestyle and has been shown to be psychometrically adequate among HIV-positive persons in a prior study (*α* = 0.67) [32]. Higher life chaos scores indicate greater stability in daily routine, the ability to plan and anticipate the future (including making appointments) and being on time. Cronbach's alpha for the Life Chaos Scale among this sample of MSM was 0.76. 

The *Alcohol Use Disorders Identification Test* (AUDIT) [33] was administered to determine whether participants were at risk for hazardous alcohol consumption or alcohol dependency. Prior research showed that 92% of persons who were diagnosed with an alcohol use disorder were classified as having harmful or hazardous alcohol use scored 8 or more on the AUDIT (the cut-off score for harmful and hazardous alcohol use) [33]. For the purposes of analysis, men with an AUDIT score between 0 and 7 were categorized as having no alcohol problem, between 8 and 14 as having a possible hazardous drinking problem, and 15 or higher as having possible alcohol dependency [34].

#### 2.3.3. Stimulant Use

Drug use was assessed by asking participants to indicate the number of times they had used any of the following 10 illicit substances in the past 30 days: codeine purchased on the street, powder cocaine, crack cocaine, amphetamines, methamphetamines, GHB, ketamine, ecstasy, heroin, or cocaine and heroin mixed together. Consistent with federal definitions of illicit stimulant drugs [6], cocaine, crack, amphetamines, methamphetamines, and ecstasy were grouped as stimulant drugs for the purpose of this study. Participants who reported any stimulant use in the past 30 days were compared to those who did not report any stimulant use in the past 30 days.

#### 2.3.4. Engagement in HIV Medical Care

Engagement in HIV medical care was assessed using a series of items, with skip patterns depending on participants' responses. First, all participants were asked “in the last 12 months, about how many medical appointments for HIV/AIDS did you make (this means all appointments you made, whether or not you missed them)?” with response options that included the following: “I have never had this kind of appointment;” “I have had this kind of appointment, but not in the last 12 months; to options from 1 to 12 appointments. Next, participants who reported scheduling 1 or more medical appointments for HIV/AIDS were asked “of the medical appointments you made for HIV/AIDS in the last 12 months, what percent of the time did you miss or cancel those appointments for any reason without rescheduling? (note: if your doctor or nurse cancelled the appointment, do not include it in your count),” with response options ranging from 0 to 100 percent. The proportion of HIV medical care appointments to those scheduled is a common measure of treatment engagement [35].

Attitudes toward engagement were assessed with two 5-point Likert-scale items. First, all participants were asked, “when thinking about all of the things you need to do and take care of in your life, how important is HIV/AIDS medical care to you?” with options from “not a priority at all” (1) to “the most important priority” (5). Second, men who attended one or more appointments for HIV in the past year were asked: “how confident are you that you can attend all of the medical appointments for HIV/AIDS that your doctor recommends in the upcoming year?” with response options ranging from 1 (very unconfident) to 5 (very confident). 

#### 2.3.5. Technology Use

To assess regular social networking website use, participants were asked to indicate which of the following websites or features they use at least once a week: *Bebo*, *The Body.com* connect bulletin boards, *Facebook*, *LinkedIn*, *MySpace*, *Poz.com* community section blogs or forums, *Xanga*, or “other” (with a write-in option). Participants were asked to report what kind of phone they had, with options including smart phone brands (defined as “a smart phone allows easy Internet browsing and may have other capabilities beyond voice calls and text messaging,” e.g., *iPhone*, *Blackberry*), a mobile phone without smart phone features, or no mobile phone.

### 2.4. Analysis

Analyses were conducted using Stata (version 12.1 for Mac) [36]. Demographic and psychosocial variables were collapsed into groups shown in Table 1. Group differences between stimulant-using and nonstimulant-using men assessed using *t*-tests (for continuous variables) or nonparametric tests (e.g., chi-square or Fisher's exact) where appropriate.

Measures of HIV medical care engagement vary considerably depending on the study [37], and new data show that annual monitoring of CD4+ T-lymphocyte may be warranted for clinically stable patients [38]. We assumed that participants must have reported scheduling and attending at least one scheduled appointment in the previous year to be considered minimally engaged. (It may be argued that scheduling and attending one HIV medical appointment in the past year is equivalent to not being engaged in HIV care. Seven men (or 3% of the sample) scheduled and attended only one HIV medical care appointment in the past year. The estimated effect of stimulant use on engagement was not altered if these 7 men were categorized as “Not in HIV Medical Care” (versus categorizing them in the “No Missed Appointments” group).) Next, men were grouped into one of three engagement in HIV medical care categories: (1) participants in the “No Missed Appointments” group reported scheduling at least 1 medical appointment for HIV in the past year and not missing any of their scheduled appointments; (2) the “Missed Appointment(s)” groups were those who scheduled two or more appointments in the past year and reported missing between 1% and 99% of those appointments; or (3) the “Not in HIV Medical Care” group included men who either (a) had not scheduled any medical appointments for HIV in the past year or (b) reported missing all of their scheduled appointments for HIV in the past year.

Responses to items assessing attitudes toward HIV medical engagement were dichotomized by grouping the responses of the two most positive response options toward HIV medical care (e.g., “a high priority” and “the most important priority”) together and grouping less positive attitudes (e.g., “not a priority at all,” “a low priority,” or “Not any more of a priority than other things in my life”) together. Differences in attitudes toward HIV medical care between stimulant-using and nonstimulant -using men were assessed using chi-square statistic. 

The effect of stimulant use in the past 30 days (two levels: yes versus no) on HIV medical care engagement (three levels: No Missed Appointments, Missed Appointment(s), and Not in HIV Medical Care) was assessed using a series of three multinomial regression models with additional blocks of variables included for each successive model: (1) an unadjusted model; (2) a model including demographic variables that were at least marginally (*P* < 0.10) associated with treatment engagement in the bivariate analyses; and (3) a model including demographic and psychosocial variables that were at least marginally associated with treatment engagement in bivariate analyses. Stata provides an option to calculate the relative risk ratio (or RRR) from the multinomial log-odds coefficient. The RRR is interpreted as the change in the outcome relative to the referent group (the “No Missed Appointments” group) for each unit change in the predictor variable given that all other variables in the model are held constant [39]. The RRR often is interpreted similarly to an odds ratio, however, used when conducting multinomial logistic regression analyses. 

Technology use variables were collapsed into those shown in Table 4 and assessed for the overall sample and by engagement in HIV medical care. Because Facebook was widely used, its use was assessed separately from other types of social networking sites. Group differences in any regular social networking site use, Facebook use, other (than Facebook) social networking site use, and mobile phone use were assessed with chi-square or Fisher's exact statistics.

## 3. Results

### 3.1. Participants

The men were on average 42 years of age and had been living with HIV for 10 years (Table 1). Most men identified as white (71%), highly educated (43% had a college degree), and experiencing significant depressive symptoms (63%). 

### 3.2. Stimulant Use

Sixteen percent (*n* = 44) of men reported using one or more types of stimulant drugs in the past 30 days. Among men reporting stimulant use, the most common was methamphetamine (54.5%), followed by cocaine (38.6%), ecstasy (20.5%), crack (18.2%), and amphetamine (18.2%). As shown in Table 1, stimulant-using men reported significantly higher life chaos scores (mean = 17.1 versus 15.1, *P* = .01) and possible alcohol dependence problems (32% versus 14%, *P* < .01) compared to nonstimulant-using men. (When alcohol use is categorized as alcohol dependency (1) versus not (0), a significantly higher proportion of stimulant-using (32%) participants continues to report alcohol dependency than nonstimulant -using participants (11%), *χ* ^2^(1, *N* = 276) = 13.50, *P* < 0.001).

### 3.3. Attitudes toward Engagement in HIV Care

Overall, most (80%) men reported that HIV medical care is a high priority (Table 2). Among men who had attended at least one HIV medical appointment in the past year, nearly two-thirds (64%) were confident that they could attend all of their HIV medical appointments in the upcoming year. 

A smaller proportion of stimulant-using men than nonstimulant-using men rated HIV medical care as a high priority (57% versus 85%; *P* < 0.01). Although a lower percentage of stimulant-using men reported high confidence to attend all of their HIV medical appointments in the upcoming year than nonstimulant-using men (54% versus 66%), this difference was not statistically different.

### 3.4. Association of Stimulant Use with HIV Treatment Engagement

Table 2 shows the proportion of men who reported no missed HIV medical care appointments, missing one or more appointments, or not attending any HIV medical care appointments in the past year. Over two-thirds (69%) of men did not miss any of their HIV medical care appointments, while 22% missed at least one, and 9% had not attended any of their appointments. The nonparametric analysis showed that stimulant-using men were more likely than nonstimulant-using men to miss one or more HIV appointments (30% versus 21%) and not attend any HIV appointments (16% versus 7%) in the past year (*P* = 0.05). 

Table 3 shows the unadjusted and adjusted effects of current stimulant use on engagement in HIV medical care in the past year. Because age (*P* = 0.01), racial/ethnic minority (*P* = 0.06), education (*P* < 0.01), depression (*P* = 0.04), and life chaos (*P* < 0.01) were associated with HIV medical care engagement in the bivariate analyses at the *P* < 0.10 level, these factors were included in Models 2 and 3. In contrast, years living with HIV, alcohol use, and CD4+ count were not associated with engagement in HIV medical care in bivariate analyses (*P* > 0.10) and, therefore, were not retained in the models.

Stimulant users were significantly more likely to report not attending any HIV medical appointments in the past year than to report not missing any of their HIV medical appointments in the unadjusted model (Model 1: RRR = 2.84, 95%CI[1.07, 7.58]), as well as in models adjusted for demographic (Model 2: RRR = 3.16, 95%CI[1.13, 8.84]) and demographic and psychosocial (Model 3: RRR = 3.44, 95%CI[1.17, 10.15]) factors (*Ps* < 0.05). In addition, nonwhite race/ethnicity was associated with not attending any HIV medical care appointments in Model 3. In contrast, stimulant use was not significantly associated with missing one or more HIV medical care appointments. However, higher levels of life chaos were significantly associated with missing one or more HIV-related medical appointments in the past year. Having a college degree was associated with a lower likelihood of not being in HIV medical care and missing one or more HIV-related appointments in the past year across all models. 

### 3.5. Technology Use

Technology and mobile phone use for the overall sample of men and by level of HIV medical care engagement is shown in Table 4. Over three quarters (78%) of men reported using a social networking site at least once a week. *Facebook* was the most commonly reported social networking site, with 61% of men reporting its use (and 42% among stimulant users). Just over one-half (54%) of men reported using one or more social networking sites other than *Facebook*, including *Poz.com* forums (31%, *n* = 85; stimulant users = 17%), *My Space* (20%, *n* = 55; stimulant users = 17%), *The Body.com* forums (12%, *n* = 33; stimulant users = 8%), *LinkdIn* (12%, *n* = 32; stimulant users = 17%), or a variety of other social networking websites (13%, *n* = 37; stimulant users = 17%). A significantly higher proportion of stimulant-using than nonstimulant-using men reported using social networking websites other than *Facebook* for the overall sample (71% versus 51%, *P* = 0.02) and among participants who had not missed any of their HIV medical appointments in the past year (75% versus 51%, *P* = 0.02). No other significant differences in social networking site use were found between stimulant users and nonstimulant users for the overall sample or within level of engagement in HIV medical care.

No significant differences were found between stimulant-using and nonstimulant-using men with respect to mobile phone use. Overall, 89% of men reported using a mobile phone. Among those who used a mobile phone, an approximately equivalent proportion used a mobile phone without smart phone features and a mobile phone with smart phone features (43% and 46%, resp.). Mobile phone use was similar among men who had missed one or more HIV medical appointment in the past year or who had not attended any HIV medical appointments, with approximately half of each group reporting using a smart phone. 

## 4. Discussion

The purpose of this study was to assess the association of stimulant use with engagement in HIV medical care and to compare technology use among stimulant-using MSM with varying degrees of HIV medical care engagement. Three findings are particularly noteworthy. First, stimulant use was significantly associated with not being in HIV medical care in both the bivariate and multivariate analyses, although not associated with greater likelihood of missing HIV-related medical appointments. Second, a high proportion of stimulant-using MSM reported that HIV medical was a low priority and had low confidence in attending all of their appointments in the upcoming year. Third, social networking website, feature, and mobile phone use were similar among men in this sample regardless of whether or not they reported recent stimulant use. Each of these findings is discussed in greater detail in the following.

Studies demonstrate that a variety of factors are associated with nonengagement or suboptimal engagement in HIV medical care, including younger age, a history of mental health problems, and a history of drug use and injection drug use [40, 41]. There is growing evidence that stimulant use has a particularly deleterious effect on the health of people living with HIV, including more rapid HIV disease progression [13]. Sixteen percent of men in this sample reported stimulant use, which is generally within the range of stimulant use reported in other national samples of MSM [42], although lower compared to MSM residing on the west coast and in the south central USA [7, 42]. The results of the current study suggest that stimulant use may exert a disruptive effect on engagement in HIV medical care. Even when factors known to be associated with greater stimulant use among people with HIV (e.g., younger age [43]) are accounted for in the models, stimulant use appears to be uniquely associated with not attending any HIV medical care appointments in this sample of MSM. In addition, and consistent with prior studies [32, 44, 45], not having a college degree, racial and ethnic minority status, and greater life chaos may place people at risk for not engaging or missing HIV-related medical appointments. These results suggest that it may be critical to assess stimulant use at time of HIV diagnosis to determine who is at elevated risk for subsequent nonengagement and, therefore, may require additional supports. 

Despite these findings, 54% of recent stimulant users report being engaged in their HIV medical care in this study. The findings of a qualitative study by Rajabiun and colleagues [46] provide some indication for what may distinguish persons with a substance use history who engage in their HIV medical care from those who do not. A high percentage of the 76 people with HIV (80%) interviewed in that study reported a lifetime history of substance use. However, those who were able to establish and maintain optimal engagement in HIV care reported greater coping and adaptive abilities than substance-using participants with suboptimal HIV medical care engagement. The results of the current study provide evidence that MSM who report current stimulant use are at elevated risk for not attending HIV medical care appointments and, in conjunction with the results from Rajabiun et al., suggest that such persons may benefit from additional coping skills and resources to engage or re-engage them in their HIV medical care. A relatively new approach may be to leverage peer navigators to assist patients at risk for default from HIV medical care [47, 48]. Facilitating and enhancing opportunities for stimulant-using persons to remain engaged in their medical care may be critical to successfully addressing deficits in the treatment cascade for HIV-positive MSM.

Beliefs and attitudes toward the medical system and engagement in medical care are known predictors of treatment engagement. In a review of 16 studies that examined African American's beliefs toward HIV medical care engagement, experiences of racism, mistrust of the medical system, and patient-provider relationship were found to impact engagement [49]. A high proportion of stimulant-using MSM in the current study reported that HIV medical care was not a high priority and that they had little confidence that they would be able to attend all of their HIV medical care appointments in the upcoming year. Heightening the prioritization of, and confidence to engage in, a specified health behavior is well recognized in a number of health behavior theories [50], as these factors are associated with the likelihood of enacting the behavior [51]. Intervention activities that heighten the prioritization of HIV medical care and confidence to enact care engagement behaviors among stimulant-using MSM may be part of a larger intervention package to link and keep this difficult-to-reach population retained in HIV care. Models of engagement in care for persons living with chronic conditions, including HIV, that acknowledge the importance of medical care prioritization and confidence have been proposed [52]. The findings of this study confirm that there may be motivational and self-efficacy deficits among stimulant-using HIV-positive MSM that may be addressed to improve retention in HIV medical care.

A number of recent technology-delivered interventions to improve the health of people living with HIV have been examined to determine the degree to which they may be acceptable or beneficial [27]. However, with few exceptions [24], technology-based interventions have typically not addressed the needs of drug-using populations [23]. In part, the lack of research into the application of technology to deliver HIV-related intervention to drug-using populations may stem from the belief that such persons do not have access to online or mobile technologies that would be required for the successful widespread dissemination of these types of interventions. However, the results of this study support the use of such technologies, as there were few differences between the proportion of stimulant-using and nonstimulant-using MSM who frequently used social network websites, features, and mobile phones. In fact, a higher percentage of stimulant-using men reported weekly use of social networking websites and features that were not Facebook than men who did not report using stimulant drugs. The comparatively high percentage of stimulant-using men in this sample who regularly used social networking websites and features and had access to mobile phones supports ongoing efforts [24] to reach stimulant-using HIV-positive MSM using these technologies. However, it is noteworthy that online social networking use was lowest among stimulant-using MSM who were not in HIV medical care. Thus, as is the case generally with not-in-treatment groups, reaching them through online social networking websites may prove the most challenging. The degree to which online social networking and mobile phone technologies may be used to reach and provide intervention to stimulant-using people with HIV should be explored further.

### 4.1. Study Limitations and Conclusions

This study has a number of limitations. First, this primarily online-recruited sample of MSM is not representative of all HIV-positive MSM or HIV-positive persons in USA. A noticeably higher percentage of respondents in this sample reported being engaged in HIV medical care compared to a nationally-representative sample of persons estimated to be retained in HIV care in the USA [3]. Discrepancies in HIV care retention findings between study samples and nationally-representative samples may be attributed to multiple factors, including inclusion criteria, sampling strategy, and varying definitions of retention in HIV medical care [53]. In addition, technology use rates may be inflated in the current sample of HIV-positive MSM given that most were recruited through online venues. Thus, the results of this study may not be generalized to other samples or populations. Second, this was a one-time cross-sectional survey that does not capture variations in stimulant and engagement in HIV medical care over time. Longitudinal data would be needed to more definitively determine the effect of stimulant use on HIV care engagement, although this was outside the scope of this study. Third, the survey was self-report, and participants actual drug use, technology use, and engagement in HIV medical care may differ from self-report due to a variety of recall and reporting biases. Thus, the results should be approached with caution and require additional confirmation from future studies. Fourth, best practices to measure and report engagement in HIV medical care have not been established [35, 53]. Although we measured engagement in HIV medical care using items described earlier and categorized men into three engagement groups, alternative measurement and categorization schemes may capture greater nuances in engagement in HIV medical care. A challenge to future research is to compare engagement and retention measures to each other and to clinical outcomes to determine a “gold standard” for measurement of these factors [53]. In addition, participants in this study may have had difficulty in estimating the percentage of missed HIV-related appointments in the prior 12 months. Finally, although a number of steps were taken to ensure that participants were valid and unique, we were not able to confirm that participants were unique respondents given that men completed the survey online.

## 5. Conclusions

Despite these limitations, this is the first study (that we are aware of) to examine the association between stimulant use and engagement in HIV medical care and to assess technology use by stimulant use and engagement in HIV care. The results provide important preliminary evidence that stimulant use negatively impacts HIV medical care engagement and that the use of a variety of emerging technologies may be a possible way to reach stimulant-using MSM. However, more research is needed to assess the acceptability of these technologies for intervention purposes and—if acceptable—best practices for adaptation of effective interventions for dissemination using technology-based applications.

## Figures and Tables

**Table 1:** Sociodemographic and psychosocial characteristics.

	Total (*n* = 276)	No stimulant use (*n* = 232)	Stimulant use (*n* = 44)	*P* value
	M^a^ (SD)^b^	M (SD)	M (SD)	

Age (in years)	42.2 (9.9)	42.5 (9.9)	40.3 (9.6)	0.18^f^
Years with HIV	9.8 (7.2)	9.6 (7.1)	11.0 (7.4)	0.25^f^
CD4+ count^c^	596.5 (318.7)^c^	571.9 (324.2)^d^	556.4 (290.4)^e^	0.78^f^
Life chaos	15.4 (4.9)	15.1 (4.7)	17.1 (5.8)	0.01^f^

	Column % (*n*)	Column % (*n*)	Column % (*n*)	

Race				
White, non-Hispanic	70.6 (195)	72.0 (167)	63.6 (28)	0.44^g^
Black	10.1 (28)	10.3 (24)	9.1 (4)	
Hispanic	15.2 (42)	13.8 (32)	22.7 (10)	
Other	4.0 (11)	3.9 (9)	4.6 (2)	
Education				
High school or less	11.2 (31)	11.2 (26)	11.4 (5)	0.34^h^
Tech school or some college	46.0 (127)	47.8 (111)	36.4 (16)	
College degree	42.8 (118)	41.0 (95)	52.3 (23)	
Depressive symptoms^i^				
No	36.4 (99)	37.1 (85)	32.6 (14)	0.57^h^
Yes	62.5 (173)	62.9 (144)	67.4 (29)	
Alcohol use^j^				
No alcohol problem	68.1 (188)	72.0 (167)	47.7 (21)	<0.01^h^
Hazardous drinking	17.8 (49)	17.2 (40)	20.5 (9)	
Alcohol dependency	14.1 (39)	10.8 (25)	31.8 (14)	

^a^Mean; ^b^standard deviation; ^c^9 missing cases, median = 524; ^d^6 missing cases, median = 527; ^e^3 missing cases, median = 522; ^f^ *t*-test; ^g^Fisher's exact test; ^h^chi-square test; ^i^using the 10-item CES-D scale [30]; ^j^using the AUDIT [33].

**Table 2:** Engagement in HIV care and attitudes toward HIV care among stimulant and nonstimulant-using men who have sex with men.

	Total	No stimulant use	Stimulant use	*P* value^a^
	Column % (*n*)	Column % (*n*)	Column % (*n*)	
Attitudes toward engagement in medical care				
*How important is HIV/AIDS medical care to you?* ^ b^				
Low priority	19.6 (54)	15.1 (35)	43.2 (19)	<0.01
High priority	80.4 (222)	84.9 (197)	56.8 (25)	
*How confident are you that you can attend all of your medical appointments for HIV/AIDS?* ^ c^				
Low confidence	36.1 (91)	34.4 (74)	46.0 (17)	0.18
High confidence	63.9 (161)	65.6 (141)	54.0 (20)	

Engagement in HIV medical care^b^				
No missed appointments	68.8 (190)	71.6 (166)	54.6 (24)	0.05
Missed appointment(s)	22.5 (62)	21.1 (49)	29.6 (13)	
Not in HIV medical care	8.7 (24)	7.3 (17)	15.9 (7)	

^a^Chi-square tests; ^b^includes full sample (*n* = 276); ^c^includes only participants who attended 1 or more HIV care appointments in past year (*n* = 252).

**Table 3:** Estimated effect of recent (past 30 days) stimulant use on engagement in HIV care in past year.

Ref. no missed appointments	Model 1^a^	Model 2^b^	Model 3^c^
	RRR^d^ (95% CI^e^), *P* value	RRR (95% CI), *P* value	RRR (95% CI), *P* value
Missed appointment(s)			
Stimulant use	1.84 (0.87, 3.87), *P* = 0.111	1.99 (0.91, 4.37), *P* = 0.085	1.73 (0.72, 4.16), P = 0.218
Age	—^f^	0.98 (0.94, 1.01), P = 0.121	0.97 (0.94, 1.01), P = 0.127
Nonwhite race/ethnicity	—	1.56 (0.82, 2.96), P = 0.173	1.68 (0.85, 3.32), P = 0.136
Education			
High school or less	—	Ref.	Ref.
Technical school/some college	—	0.45 (0.19, 1.08), P = 0.075	0.51 (0.20, 1.28), P = 0.152
College degree	—	0.20 (0.08, 0.52), P = 0.001	0.21 (0.08, 0.58), P = 0.003
Depression	—	—	1.04 (0.48, 2.27), P = 0.915
Life chaos	—	—	1.17 (1.09, 1.26), P = 0.000

Not in HIV medical care			
Stimulant use	2.84 (1.07, 7.58), *P* = 0.036	3.16 (1.13, 8.84), P = 0.028	3.44 (1.17, 10.15), P = 0.025
Age	—	0.97 (0.92, 1.01), P = 0.152	0.97 (0.92, 1.01), P = 0.162
Nonwhite race/ethnicity	—	2.33 (0.95, 5.70), P = 0.063	2.58 (1.04, 6.40), P = 0.041
Education			
High school or less	—	Ref.	Ref.
Technical/some college	—	0.64 (0.17, 2.31), P = 0.491	0.73 (0.20, 2.73), P = 0.642
College degree	—	0.22 (0.05, 0.94), P = 0.040	0.22 (0.05, 0.98), P = 0.047
Depression^g^	—	—	0.54 (0.19, 1.52), P = 0.242
Life chaos^h^	—	—	1.11 (0.99, 1.23), P = 0.065

Notes: ^a^unadjusted model; ^b^Model 1 plus demographic variables significantly associated with treatment engagement in the bivariate analyses; ^c^Model 2 plus psychosocial variables significantly associated with treatment engagement in the bivariate analyses, 5 missing cases; ^d^relative risk ratio; ^e^confidence interval; ^f^variable not included in model; ^g^using the 10-item CES-D scale [30]; ^h^using the life chaos scale [32].

**Table 4:** Technology use by stimulant use and engagement in HIV care.

	Total	No stimulant use	Stimulant use	*P* value
	Column % (*n*)	Column % (*n*)	Column % (*n*)	
*Total (n = 276) *		*n = 232 *	*n = 44 *	
Any social network site use	77.5 (214)	76.7 (178)	81.8 (36)	0.46^b^
Social network site used				
Facebook	60.9 (168)	62.1 (144)	55.6 (24)	0.35^b^
Other	54.0 (149)	50.9 (118)	70.5 (31)	0.02^b^
Mobile phone^a^				
No mobile phone	11.3 (31)	12.1 (28)	6.82 (3)	0.67^c^
Mobile phone	42.6 (117)	42.0 (97)	45.5 (20)	
Smart phone	46.2 (127)	45.9 (106)	47.7 (21)	

*No missed appointments (n = 190) *		*n = 166 *	*n = 24 *	
Any social network site use	77.9 (148)	76.5 (127)	87.5 (21)	0.30^c^
Social network site used				
Facebook	60.5 (115)	60.8 (101)	58.3 (14)	0.81^b^
Other	53.7 (102)	50.6 (84)	75.0 (18)	0.03^b^
Mobile phone^a^				
No mobile phone	8.5 (16)	9.1 (15)	4.2 (1)	0.80^c^
Mobile phone	47.1 (89)	47.3 (78)	45.8 (11)	
Smart phone	44.4 (84)	43.6 (72)	50.0 (12)	

*Missed appointment(s) (n = 62) *		*n = 49 *	*n = 13 *	
Any social network site use	82.3 (51)	81.6 (40)	84.6 (11)	1.00^c^
Social network site used				
Facebook	66.1 (41)	69.4 (34)	53.9 (7)	0.29^b^
Other	61.3 (38)	57.1 (28)	76.9 (10)	0.34^c^
Mobile phone				
No mobile phone	14.5 (9)	16.3 (8)	7.7 (1)	0.43^c^
Mobile phone	37.1 (23)	32.7 (16)	53.9 (7)	
Smart phone	48.4 (30)	51.0 (25)	38.5 (5)	

*Not in HIV medical care (n = 24) *		*n = 17 *	*n = 7 *	
Any social network site use	62.5 (15)	64.7 (11)	57.1 (4)	1.00^c^
Social network site used				
Facebook	50.0 (12)	52.9 (9)	42.9 (3)	1.00^c^
Other	37.5 (9)	35.3 (6)	42.9 (3)	1.00^c^
Mobile phone				
No mobile phone	25.0 (6)	29.4 (5)	14.3 (1)	0.85^c^
Mobile phone	20.8 (5)	17.7 (3)	28.6 (2)	
Smart phone	54.2 (13)	52.9 (9)	57.1 (4)	

Notes: ^a^1 missing case; ^b^chi-square test; ^c^Fisher's exact test.
